# Pneumonia partly mediates the detrimental effect of *APOE4* on AD risk: New insights from Causal Mediation Analysis

**DOI:** 10.1002/alz70861_109005

**Published:** 2025-12-23

**Authors:** Svetlana Ukraintseva, Rachel Holmes, Hongzhe Duan, Deqing Wu, Konstantin Arbeev, Anatoliy Yashin

**Affiliations:** ^1^ Duke University, Durham, NC USA

## Abstract

**Background:**

*APOE4* and its proxy, rs2075650 (G) allele, are strongly associated with Alzheimer’s disease (AD), though the mechanisms remain debated. *APOE4* has also been linked to infection severity. Infections, including pneumonia, have, in turn, been associated with AD risk. We hypothesize that the detrimental effects of *APOE4* and rs2075650 (G) on AD occurrence may be mediated through pneumonia.

**Method:**

We performed a Causal Mediation Analysis (CMA) using the Framingham Heart Study Original Cohort (FHS) and Cardiovascular Health Study (CHS) data. Carrying status (yes vs. no) of *APOE4* or rs2075650 (G) was used as “treatment”. Pneumonia onset vs. no pneumonia before age 83 served as the binary mediator. The binary AD outcome was defined as: (0) AD onset at any age vs. (1) no AD in those who survived to age 85 or beyond. A total of 726 individuals from FHS and 1,450 from CHS were included. SAS procedure PROC CAUSALMED under counterfactual framework was used, controlling for covariates. Log link was applied to address the non‐rare outcome situation. Mediation effects were evaluated using the total effect (TE) and percentage of the TE mediated by pneumonia (PM).

**Result:**

Pneumonia onset before age 83 was a significant mediator of detrimental effects of *APOE4* and rs2075650 (G) on AD occurrence in the total sample and in subsamples stratified by sex. In CHS, rs2075650 (G) carriers were more than twice as likely to develop AD at any age (TE=2.11‐2.16; *p* ‐value=0.0001‐0.0002), compared to non‐carriers, when considering the effect of mediator. In FHS, *APOE4* carriers were about 32% more likely to develop AD at any age compared to non‐carriers (*p* ‐value=0.012‐0.013). The PM was between 13%‐18% in the total and stratified FHS samples (*p* =0.017‐0.079), and around 10% in CHS (*p* ‐value=0.077‐0.106).

**Conclusion:**

Findings of our CMA suggest that *APOE4* and rs2075650 (G) carriers may have an increased risk of AD, partly due to their higher likelihood of developing pneumonia compared to non‐carriers. These findings broadly align with the idea that the detrimental effects of *APOE4* on AD may involve higher vulnerability to certain infections, which in turn may contribute to brain damage and neurodegeneration.

